# A review of extraction, purification, structural properties and biological activities of legumes polysaccharides

**DOI:** 10.3389/fnut.2022.1021448

**Published:** 2022-10-05

**Authors:** Yingying Zhu, Xuewei Feng, Jianhang Guo, Li Wang, Xudan Guo, Xiangzhen Zhu

**Affiliations:** ^1^Henan Key Laboratory of Cold Chain Food Quality and Safety Control, College of Food and Bioengineering, Henan Collaborative Innovation Center for Food Production and Safety, Zhengzhou University of Light Industry, Zhengzhou, China; ^2^State Key Laboratory of Cotton Biology, Institute of Cotton Research, Chinese Academy of Agricultural Sciences, Anyang, China; ^3^Basic Medical College, Hebei Higher Education Institute Applied Technology Research Center on TCM Formula Preparation, Hebei TCM Formula Preparation Technology Innovation Center, Hebei University of Chinese Medicine, Shijiazhuang, China

**Keywords:** legumes, polysaccharides, extraction methods, structural characterization, biological activity

## Abstract

In recent years, polysaccharides derived from legumes polysaccharides have aroused worldwide interests. Phytochemical and pharmacological studies have studied the physicochemical properties (emulsification, stability and foaming) and demonstrated the biological activities (immune regulation, anti-oxidation, anti-tumor, hypoglycemic, hypolipidemic and intestinal flora regulation) of legumes polysaccharides. Besides, it is reported that the extraction methods will affect the structural features of polysaccharides, thus further changing their physicochemical properties and biological activities. This review appraised the available literatures described the extraction, purification, structural characterization, biological activity and functional properties of legumes polysaccharides in recent years. It can provide useful research underpinnings and updated information for the development and application of related polysaccharides in functional food and medicinal field.

## Introduction

Legumes are a large group with pods as a common feature. They are the third largest family of angiosperms, with about 690 genera and more than 17,600 species, widely distributed all over the world. Legumes mainly include soybeans, black beans, adzuki beans, mung beans, lentils, cowpeas, kidney beans, peas, chickpeas, etc. Modern chemical and pharmacological studies have shown a positive correlation between the consumption of legumes and a reduced risk of Chronic metabolic diseases, such as cardiovascular disease, diabetes, obesity and cancer (1, 2). This is related to the fact that legumes are rich in polysaccharides, protein, vitamins, minerals, unsaturated fatty acids, phenolic compounds and other beneficial nutritional factors (3).

In recent years, the research on legumes has mainly focused on protein, but with the rapid development of glycochemistry and glycobiology technology, legumes polysaccharide has gradually become a research hotspot of scholars worldwide (4, 5). Legumes polysaccharides obtained by different extraction and purification methods have different physicochemical properties and biological activities, including immune regulation, anti-oxidation, anti-tumor, hypoglycemic, gastrointestinal protection and so on (6–10). Additionally, legumes polysaccharides have physical and chemical properties such as emulsifying and stability, which can improve the quality and appearance of dairy products (11, 12).

However, throughout the available literatures, there has been no systematic review of legumes polysaccharides, neither on the extraction and purification methods nor on the structural characterization and biological activities. Moreover, legumes polysaccharides are widely used and can be used in milk beverages, flour products, functional foods and health care products. Therefore, this paper systematically reviews the extraction, isolation, purification, structural characteristics, biological activities and functional properties of legume polysaccharides, which lays a foundation for promoting the research of legumes polysaccharides in the food industry and biomedicine.

## Extraction, isolation and purification methods

**Figure 1:**
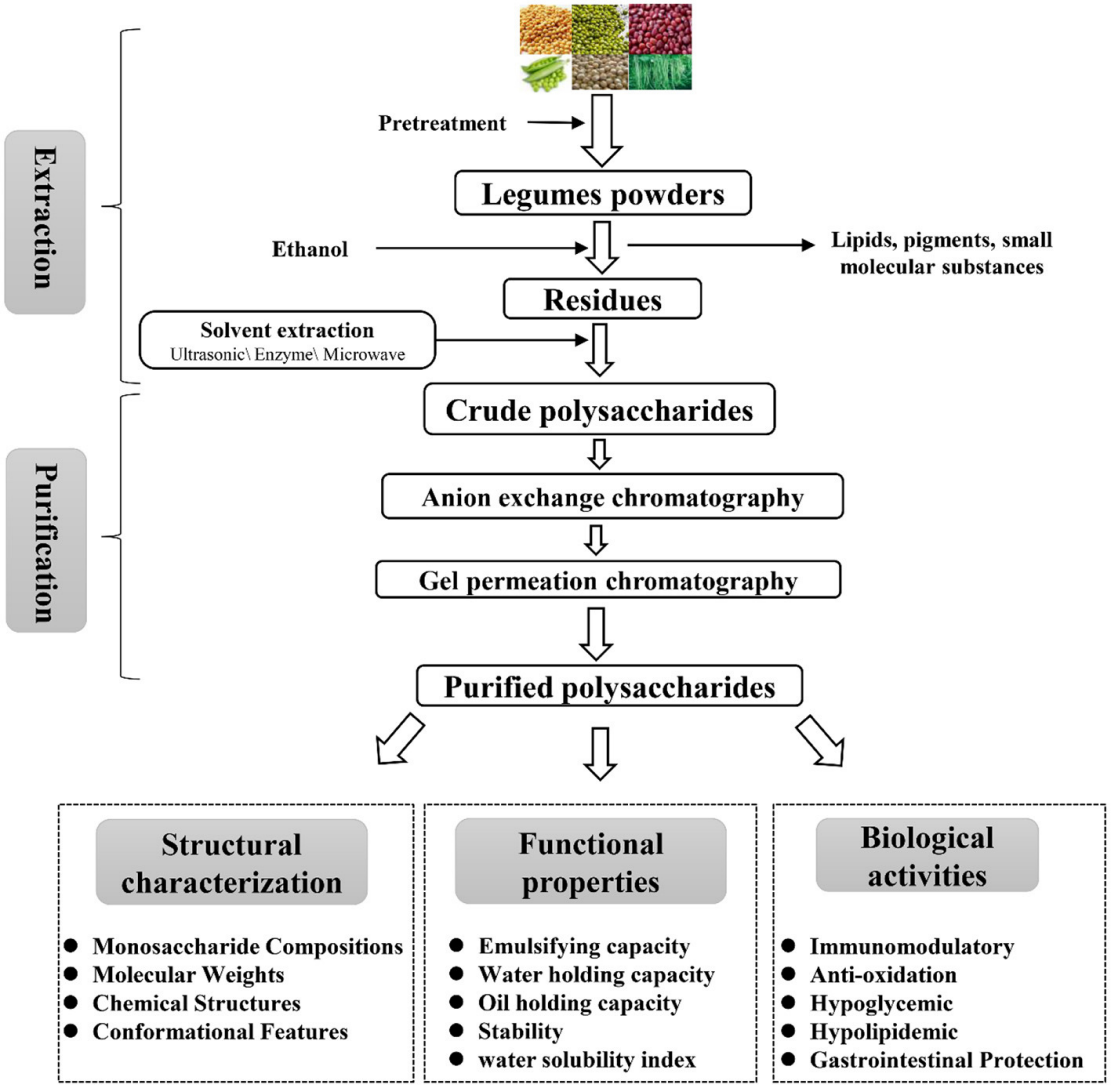
Schematic representation of the extraction, purification, and bioactivity of polysaccharides from legumes.

Legumes polysaccharides are structural components of the plant cell wall, so extraction methods are usually based on the deconstruction of the cell wall. The extraction method is to dissolve the polysaccharide from the cell wall under moderate extraction conditions without changing their structure of the legume's polysaccharides (7, 13, 14). The extraction and purification of legumes polysaccharides are shown in Figure 1 and Table 1. First, the legumes were dried, crushed, and passed through a 50-mesh sieve; then, the powder was soaked in 95% ethanol for 3 h to remove pigments, lipids, oligosaccharides and other small molecular substances (23); next, the dry legumes powder was extracted with hot water at 60–95°C, centrifuged (3,000–5,000 rpm), the supernatant was collected, concentrated, and four times the volume of absolute ethanol was added, overnight at 4°C to precipitate the legumes polysaccharide; finally, the crude legumes polysaccharides were redissolved, deproteinized, decolorized, dialyzed, and freeze-dried to obtain the legumes polysaccharide (18, 24). At present, the extraction methods of legumes polysaccharides are mainly divided into hot water extraction, acid-base extraction, enzymatic extraction, ultrasonic-assisted extraction, microwave-assisted extraction, hot-compressed water extraction and subcritical extraction (12, 22, 25, 26).

**Table 1:** A summary of the extraction of legumes polysaccharides.

**Types**	**Time (h)**	**Solid-liquid ratio**	**Temperature (°C)**	**Solvent**	**Other conditions**	**Yield (%)**	**References**
BSPS	6.4	1:20	92	Water	3 times	2.56	(15)
SHP	2	1:10	65	Water	1–3 times	9.2	(6)
BSCP	2.22	1:22.3	100	Water	1 time	10.56	(16)
A-SSCP	0.33	1:20	85	0.6% (w/v) ammonium oxalate	Microwave power 480 W	9.3	(7)
AAP	4	1:20	90	Alkali solution	0.3% (w/w) NaBH_4_ 2 times	3.28	(17)
MWP	4		90	Water	Ultrasonic power 700 W 2 times	3.25	(18)
MEMP	0.019	1:17		Water	Microwave power 700 W	6.003	(19)
MSP	0.5	1:20	25	Water	Ultrasonic power 150 W 2 times	7.6	(20)
MBP-1	3	1:20	55	Water	(2%, w/w) cellulase	1.61	(21)
sCW	0.5	1:12	120	0.2% aqueous citric acid	Subcritical water extraction pressure 50 bra	11.8	(22)

Hot-water extraction is most commonly used for laboratory extraction of polysaccharides and is also widely used in industrial extraction processes. Liu et al. (15) used response surface methodology to optimize the extraction conditions of black soybean polysaccharides: extraction temperature 92°C; extraction time 6.4 h; ratio of water to material of 20 ml/g. Under these conditions, the extraction yield of three rounds was 2.56%. Yao et al. (17) first used hot water extraction and then alkali extraction, increasing the extraction temperature of polysaccharides, and the polysaccharide yield increased to 9.59%. In order to improve the yield of polysaccharides, methods such as microwave and ultrasound are also used to assist extraction (7, 20, 27). Maryanne et al. (28) optimized ultrasonic-assisted extraction of pea polysaccharide using response surface methodology. The optimal extraction conditions were: ultrasonic power 135.34 W, extraction time 48.61 mins, extraction temperature 68.25°C and ratio of water to raw material of 33.6:1, here conditions, the polysaccharide extraction was 7.37%. Han et al. (7) compared the extraction of soybean hull polysaccharides by hot water extraction, microwave-assisted ammonium oxalate extraction and microwave-assisted sodium citrate extraction. Among them, the microwave-assisted ammonium oxalate extraction method had the highest yield (9.3%), the microwave-assisted sodium citrate method had the second highest yield (3.6%), and the hot water extraction method had the lowest yield (1.2%). Ultrasound and microwave can damage the cell wall, cause the outflow of intracellular substances, and improve the extraction efficiency of polysaccharides, but long-term use can lead to structural damage of polysaccharide (29). In addition to physical extraction, enzymatic extraction of legumes polysaccharides is also used. Enzymes can change the permeability of the cell wall and dissolve the cell contents, thereby increasing the extraction efficiency (30). Jiang et al. (21) used cellulase to assist the extraction of mung bean skin polysaccharide, and the extraction rate was 1.61%, which was much higher than that of hot water extraction.

To sum up, the hot water extraction method is simple and convenient, but the extraction rate is low; both ultrasonic-assisted and microwave-assisted extraction methods can improve the extraction efficiency, but long-term ultrasonic or microwave irradiation will change the polysaccharide structure; enzyme-assisted extraction method has the advantages of mild reaction and high extraction efficiency, but the production cost is high.

It is reported that soybean polysaccharide is a heteropolysaccharide containing different components. In order to determine the composition and structure of legumes polysaccharide, it is necessary to further purify the crude polysaccharide. At present, polysaccharide purification methods mainly include fractional alcohol precipitation, ultrafiltration and column chromatography (31, 32). Among them, the fractional alcohol precipitation and ultrafiltration are the preliminary separation, and then the column chromatography is used for further purification. Column chromatography can be divided into anion exchange chromatography and gel permeation chromatography. Anion chromatography columns (DEAE-Cellulose and DEAE-Sepharose Fast Flow) separate acidic and medium polysaccharides based on polysaccharide polarity. Gel permeation chromatography (Sephadex-G series and Sephacryl-S series) separates polysaccharides based on molecular weight differences (27, 31, 33). For example, Wang et al. (6) used different concentrations of NaCl to separate three different polysaccharides (F1, F2, F3) by DEAE Sepharose Fast Flow. Hu et al. (34) used DEAE-Cellulose to separate neutral polysaccharides (SSPS-N) and acidic polysaccharides (SSPS-A) from Okara. Ye et al. (35) used DEAE Fast Flow and Sephadex G-100 to separate and purify CHPS-1, CHPS-2 and CHPS-3 with molecular weights of 3.1 × 10^6^, 1.5 × 10^6^ and 7.8 × 10^5^ Da from chickpea hull polysaccharides, respectively. Zhang et al. (36) isolated two fractions, W-DE-GPP and N-DE-GPP, from pea, using DEAE Fast Flow and Sephadex G-100 to separate and purify two main components.

## Physiochemical and structural features

The structure of plant polysaccharides is diverse and complex, and the biological activities of polysaccharides with different structures are also different. Generally, the structural characteristics of plant polysaccharides mainly include monosaccharide composition, molecular weight, chemical structure, and spatial conformation (27). The homogeneous legumes polysaccharides obtained by separation and purification can be obtained by acid hydrolysis, methylation analysis, periodate oxidation, Smith degradation, infrared spectroscopy (IR), gas chromatography-mass spectrometry (GC-MS), nuclear magnetic resonance (NMR), high performance liquid chromatography (HPLC), gas chromatography (GC), scanning electron microscope (SEM), atomic force microscope (AFM), X-ray diffraction (XRD) and other instrumental analysis methods are used to determine the basic chemical structure (5, 30, 31, 37). The structural characteristics of legumes polysaccharides such as monosaccharide composition, molecular weight, chemical structure and biological activity are summarized in Table 2.

**Table 2:** Legumes polysaccharide: source, chemical structure, biological activity.

**No**.	**Compound name**	**Source**	**Mw (Da)**	**Monosaccharide composition**	**Structures**	**Biological activities**	**References**
1	BSPS-1	Black soybeans	1.95 × 10^5^	Ara, Rha, Gal, Glu, and Man in the molar ratio of 1.79:1.00:2.59:26.54:1.01.	Backbone consisting of α-(1 → 6)-D-Glc*p* residues		(38)
2	BSPS-3	Black soybeans	1.88 × 10^5^	Ara, Rha, Gal, and Man in the molar ratio of 16.80:3.60:33.66:1.00	Backbone consisting of β-(1 → 3)-D-Gal*p* residues, branch consisting of α-(1 → 5)-L-Ara*f, α*-(1 → 2)-L-Rha*p* and *O*-Me-β-(1 → 4)-D-GlcA*p* residues		(38)
3	BSCP-1	Black soybean	7.55 × 10^5^	Gal, Man, Rha in the ratio of 6.01:3.56:1.00.		Anti-cancer	(16)
4	SSPS-N-b	Okara	8.6 × 10^3^	Glu, Gal, Man in the ratio of 36.6:34.6:26.6	Backbone consisting of β-(1 → 4)-D-Man and β-(1 → 4)-D-Glu residues	Antioxidant	(34)
5	PMP	Soybean curd residue	3.07 × 10^6^	Rha, Ara, Xyl, Man, Glu, and Gal in the ratio of 0.08:0.25:0.16:0.07:0.28:1.00		Antioxidant	(8)
6	P_A_-150	Soy hulls		Ara, Gal, Glu, Xyl, Man in the ratio of 39.2:25.9:30.7:2.1:2.1	Backbone consisting of α-L-Arabinofuranosyl, 4-*O*-Me-α-D-Glc*p*A and α-D-Gal		(26)
7	SHP-1	Soy hull	4.85 × 10^5^	Gal*p*A, Gal, Rha Ara in the ratio of 46.5:17.95:14.77:13.97	Backbone consisting of α-(1 → 4)-D-Gal*p*A, α-(1 → 2)-L-Rha*p, β*-(1 → 4)-D-Gal, α-(1 → 2)-L-Ara residues		(39)
8	AWP-2	Adzuki beans	2.96 × 10^4^	Ara, Man, Gal, Glu in the ratio of 0.4:2.6:5.3:0.7.		Antioxidant, Immunoregulation	(17)
9	MBP-2	Mung bean	2.08 × 10^5^	Rha, Ara, Gal, Glc, Xyl, Man, GalA in the ratio of 0.15:0.67:0.29:3.64:0.22:0.12:1.11		Antioxidant, antibacterial	(21)
10	WPUCP	Chickpeas	7.68 × 10^5^	Gal, Ara, Rha, GalpA	Backbone consisting of β-(1 → 4)-D-Gal*p, α*-(1 → 5)-L-Ara*f* residues	Antioxidant	(40)

### Monosaccharide compositions

Monosaccharide composition is the most basic and core of the study of plant polysaccharide structure. In most cases, the glycosidic bonds in polysaccharides are completely destroyed by acid hydrolysis, followed by derivatization, and the derivatives are qualitatively and quantitatively analyzed by GC, GC-MS, HPLC (5, 13, 41). Due to the different varieties, sources and extraction and purification processes of legumes, the monosaccharide components extracted from legumes polysaccharides are different, but the polysaccharides are composed of glucose (Glu), galactose (Gal), arabinose (Ara), mannose (Man), xylose (Xyl) and different uronic acids in different molar ratios, and the composition and molar ratio of different monosaccharides were related to the sources and extraction methods of legumes polysaccharides (3). According to Table 2, black soybean polysaccharides were mainly composed of glucose and galactose; soybean polysaccharides were mainly composed of glucose, galactose and arabinose; adzuki bean polysaccharides were mainly composed of galactose and mannose; mung bean polysaccharides were mainly composed of glucose and galactose. For example, Liu et al. (15) used DEAE-52 and Sepharose CL-4B to separate and purify three component polysaccharides (BSPS-1, BSPS-2, BSPS-3) from black soybean. Chemical analysis indicated that BSPS-1 was composed of arabinose, rhamnose (Rha), galactose, glucose and mannose in the molar ratio of 1.79:1.00:2.59:26.54:1.01; BSPS-2 was composed of arabinose, rhamnose, xylose, galactose and mannose in the molar ratio of 8.10:4.80:9.15:13.38:1.00; BSPS-3 was composed of arabinose, rhamnose, galactose and mannose in the molar ratio of 16.80: 3.60:33.66:1.00. Meanwhile, Yao et al. (17) compared the effects of different extraction methods on the monosaccharide composition of adzuki bean, and found that the monosaccharide composition of the water extraction component was mainly composed of rhamnose, arabinose, mannose, galactose and glucose; the alkaline extraction components are mainly composed of rhamnose, arabinose, mannose, galactose and galacturonic acid.

### Molecular weights

At present, the measurement methods of molecular weight mainly include osmotic pressure method, viscosity method, sedimentation method and HPLC, especially high-performance gel permeation chromatography (HPGPC) is the most widely used method for molecular weight measurement (29, 42). The study found that the molecular weight of legumes polysaccharides is generally between 10^3^ and 10^6^ Da, and polysaccharides with higher molecular weights tend to have stronger physicochemical properties. Meanwhile, different extraction methods have a great impact on the molecular weight of soy polysaccharides, such as long-term ultrasonic or microwave treatment will destroy the polysaccharide molecular chain, resulting in the fragmentation of the polysaccharide main chain and reducing the molecular weight of the polysaccharide (43). Two polysaccharide fractions with immunomodulatory activity (MWP-1, MWP-2) were isolated from mung bean and their molecular weights were 68.4 and 52.4 kDa (18). In addition, two kinds of polysaccharides with molecular weights of 139 and 208 kDa were extracted from mung bean husk by enzyme extraction method and hot water extraction method, respectively. It was found that the polysaccharide with larger molecular weight was more stable in structure and stronger in activity (21). Nomura et al. (44) compared the effect of different pH on lentil polysaccharides, and the molecular weight of polysaccharides under acidic conditions (830 kg/mol) was lower than that under neutral conditions (1,130 kg/mol).

### Chemical structures

**Figure 2:**
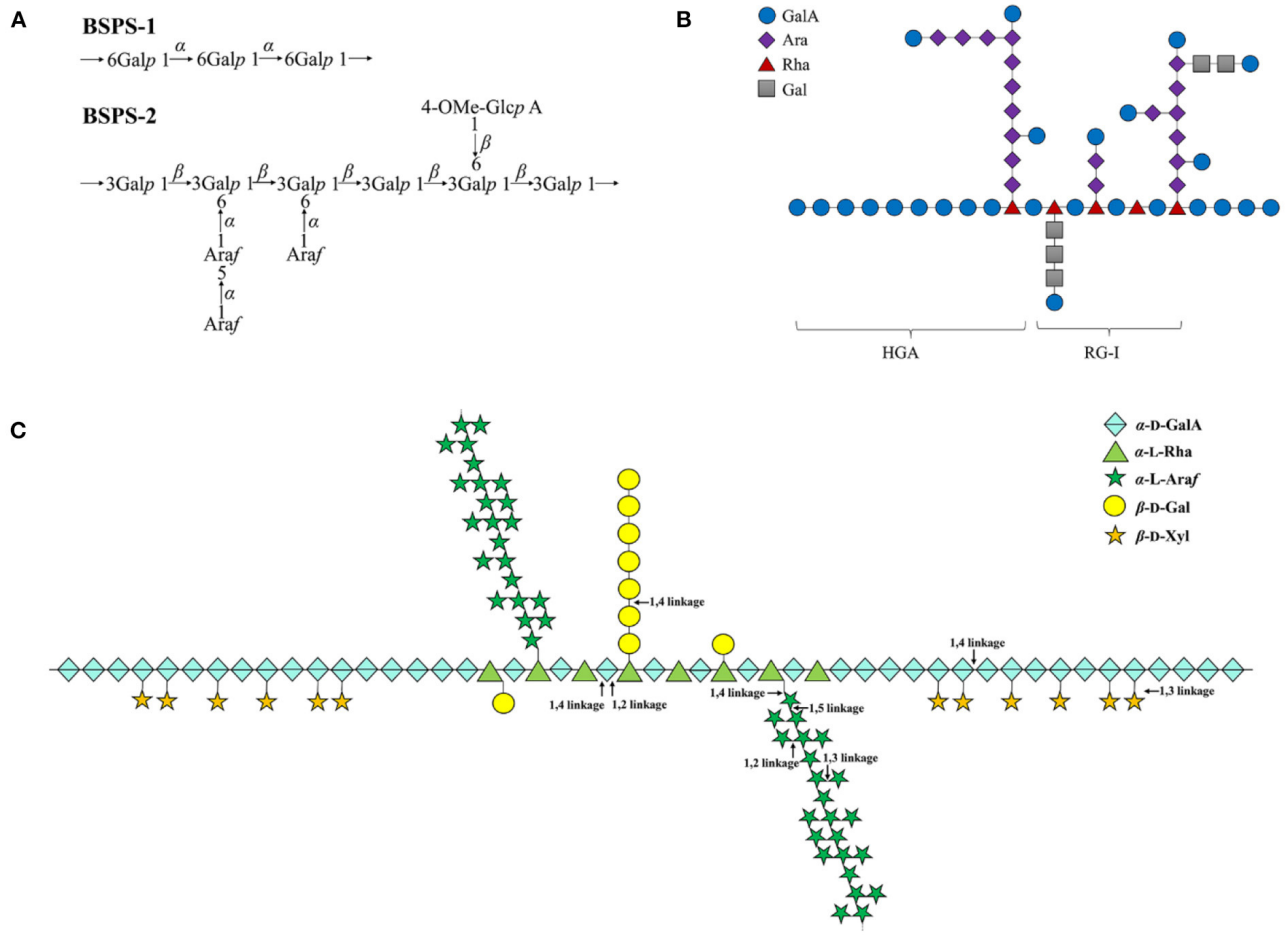
**(A–C)** The chemical structure of legumes polysaccharides. 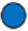, GalA; 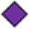, Ara; 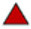, Rha; 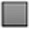, Gal; 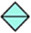, α-D-Gal; 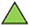, α-L-Rha; 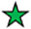, α-L-Araf; 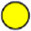, β-D-Gal; 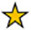, β-D-Xyl; HGA, a smooth region; RG-I, a hairy region.

In addition to the study of monosaccharide composition and molecular weight of legumes polysaccharide, the chemical structure of polysaccharide is also the focus of research (Table 2). The chemical structures of polysaccharides were obtained by methylation analysis, Smith degradation, IR, NMR and other methods (23). So far, chemical structures of legumes polysaccharides have been reported (Figure 2). Liu et al. (38) analyzed the structures of the two purified polysaccharide components (BSPS-1 and BSPS-2). According to the methylation analysis and NMR results, the possible structural units of the polysaccharides were speculated as shown in Figure 2A. They found that BSPS-1 is produced by 1,6-α-D-glucopyranosyl residues, which is a linear (1 → 6)-α-D-glucan; whereas BSPS-3 is mainly composed of a 1,3-β-D-galactopyranosyl residue backbone with side chains substituted at the *O*-6 position consisting which is a type II arabinogalactan.

Yang et al. (39) used microwave-assisted ammonium oxalate to extract a novel acidic polysaccharide (SHP-1) with a molecular weight of 4.81 × 10^5^ g/mol from soybean. It is speculated that its chemical structure is that type I pectin is composed of 2/3HG and 1/3RG-I as shown in Figure 2B. It is divided into a smooth region (HGA) and a hairy region (RG-I), and the smooth region consists of α-D-galacturonic acid residue through a 1 → 4 glycosidic bond in linear homogalacturonans; the hairy region was highly branched, and replaced by galactan, arabinan or arabinogalactan.

Noguchi et al. (45) used pectinase and biochemical methods to analyze the structure of pea pectin polysaccharide, and deduced the structure of pea pectin polysaccharide as shown in Figure 2C. Pea pectin polysaccharide contains 50% arabinose, and the neutral sugar side chain is attached to the rhamnose residue of rhamnogalacturonan-I (RG-I). The RG-I backbone was calculated to contain multiple repeats of [ → 2)-α-L-Rha-(1 → 4)-α-D-GalA-(1 → ]. Galactose and galactooligosaccharides were attached to ~35% of the rhamnose residues in RG-I. Long β-(1 → 4)-galactan was also present as the side chains. Considering the influence of neutral sugars in the main chain, the molar ratio of RG-I, XGA and HG was 7:1:2.

Goff et al. (46) isolated a polysaccharide component composed of galacturonic acid and xylose from pea, and the methylation results showed that the polysaccharide backbone was composed of α-(1 → 4) galacturonan, the side chain consists of (1 → 2) xylosyl residues.

### Conformational features

So far, there have been a lot of studies on the primary structure of legumes polysaccharides, but few reports on the chain conformation (43). At present, the advanced structure characterization of polysaccharide mainly includes SEM, AFM, XRD, Congo red (5, 14). For example, the polysaccharide (PMP) extracted from legumes dregs has a smooth surface under SEM and AFM, showing an irregular loose sheet-like structure. In the Congo red experiment, the polysaccharide and Congo red formed a complex, and there was an obvious red shift phenomenon, indicating that PMP has a triple helix structure (8). Guo et al. (47) extracted leguminous polysaccharide (CHPS) from chickpea shells by hot water extraction. The surface of CHPS was loose and porous under SEM, and there was a dispersion peak under XRD, thus confirming that CHPS is semi-crystalline in nature.

## Biological activities

In recent years, with the in-depth research on legumes polysaccharides, it has been found that has good biological activities. For example, it can prevent hypertension, diabetes, and heart disease. In addition, it also has certain effects in antioxidant, immune regulation, gastrointestinal protection, anti-tumor, and antibacterial (2, 3). A large number of experiments have shown that polysaccharides are important biologically active components in legumes, and the biological activity is closely related to the structure of polysaccharides (43).

### Immunomodulatory

**Figure 3:**
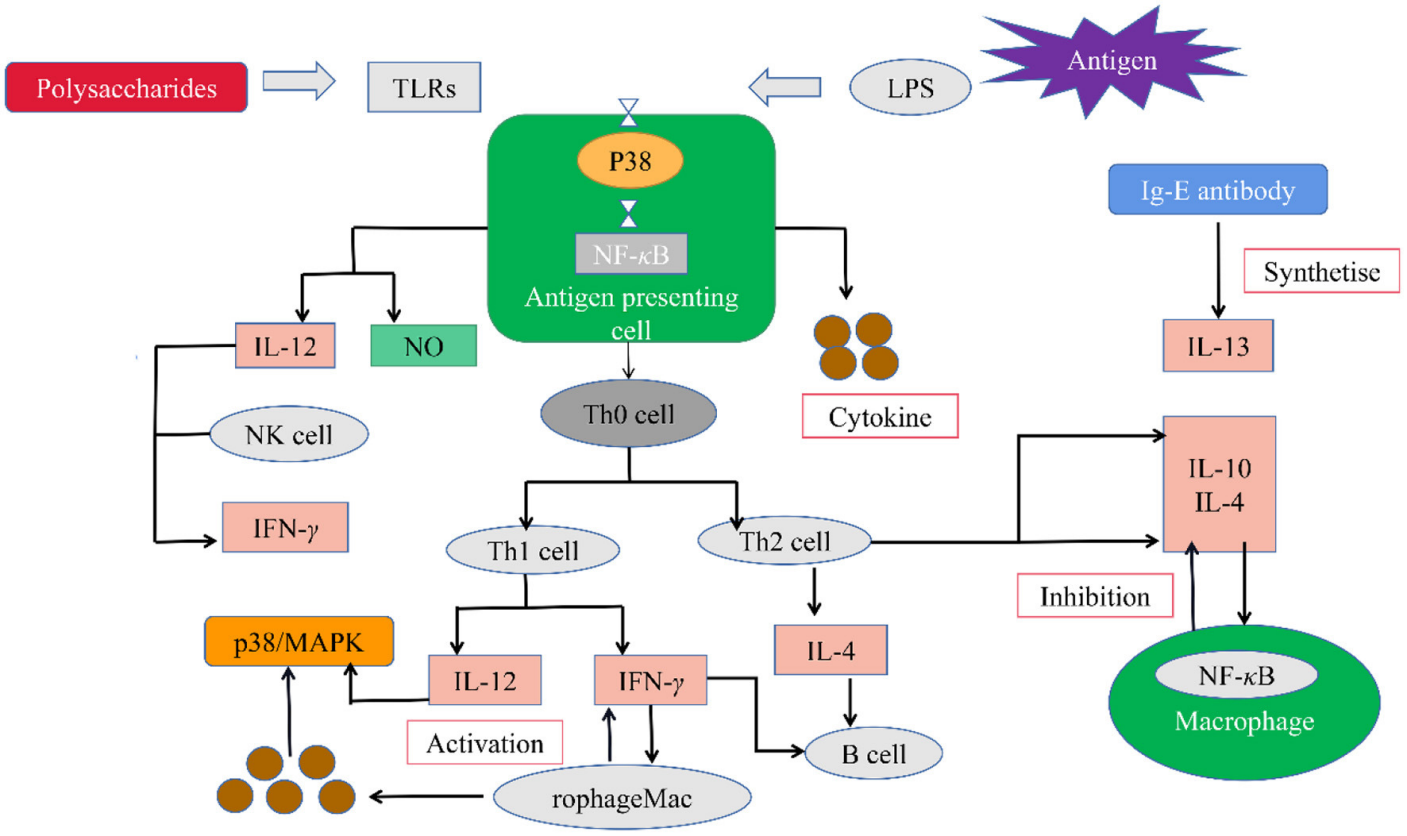
The potential immunomodulatory mechanisms of legumes polysaccharides.

Immunomodulation is the most important biological activity of plant polysaccharides and an important defense strategy against infection, inflammation and cancer (32). Polysaccharides play an immune role mainly by enhancing the functions of natural killer cells, lymphocytes, T-cells, B-cells, macrophages and other immune cells (48). The schematic diagram of the immunomodulatory mechanism of polysaccharides is shown in Figure 3. Polysaccharides combine with TLRs to promote antigenic cells to release cytokines such as interleukin-2 (IL-2), interleukin-6 (IL-6), interferon-γ (IFN-γ), tumor necrosis factor-α (TNF-α), nitric oxide (NO) (42).

Wang et al. (6) investigated the immune activity of soybean chitin polysaccharide (SHP) on macrophages. The study found that SHP can promote the proliferation of RAW 264.7 cells, produce NO, IL-6, TNF-α, which was specifically recognized by Toll-like receptor 2, and then up-regulate the expression levels of mitogen-activated protein kinase and nuclear factor κB pathway downstream proteins.

In one case, three novel polysaccharides (AWP-1, AWP-2, AAP-1) were isolated from adzuki bean, all of which stimulated macrophages to produce NO in a dose-dependent manner, but AWP-2 had the strongest activation effect on macrophages. At the same time, AWP-2 can promote the production of TNF-α and IL-6 in RAW 264.7 macrophages, and enhance the anti-inflammatory and anti-tumor activities (17). Similarly, two polysaccharides (MWP-1, MWP-2) with molecular weights of 68.4 kDa and 52.4 kDa were isolated from mung bean, which could stimulate mouse macrophages to secrete NO, TNF-α and IL-6 in a concentration-dependent manner (18).

Studies have found that polysaccharide structure such as molecular weight, chemical structure, and monosaccharide composition can have a huge impact on the immunomodulatory activity of polysaccharides (43). For example, Ketha et al. (49) studied the immune activity of mung bean non-starch polysaccharides and found that molecular weight, uronic acid, arabinose, galactose and type II arabinogalactan content appeared to play a key role in macrophage activation.

### Anti-oxidation

Oxidative stress and imbalance of free radicals in the body are the main factors leading to the occurrence of diseases such as aging, inflammation, and cancer (32). The antioxidant capacity of legumes polysaccharides is usually evaluated by various free radical tests, such as *in vitro* DPPH free radical, OH free radical, ABTS^+^ free radical, O^2−^radical scavenging ability.

For example, Hu et al. (34) isolated different components of polysaccharides (SSPS, SSPS-N, SSPS-A) from bean dregs, and found that their ability to scavenge DPPH radicals, OH radicals, and O^2−^radicals was concentration-dependent. Both SSPS and SSPS-A could scavenge DPPH radicals, OH radicals and O^2−^radicals, and the most significant concentration was 10 mg/ml, while SSPS-N could only scavenge DPPH radicals and O^2−^radicals. Wang et al. (19) isolated two polysaccharides from mung bean skin by microwave-assisted hot water extraction. MEMP-1 is mainly composed by mannose and galactose, while MEMP-2 is mainly composed of rhamnose and galactose. Antioxidant results showed that both polysaccharides had strong hydroxyl radical and DPPH radical scavenging activities, among which MEMP-2 had the highest antioxidant activity. This may be due to the differences in the composition and molecular weight of monosaccharides, which lead to different functions of polysaccharides. Meanwhile, Jalili Safaryan et al. (28) used ultrasound-assisted hot water to extract pea polysaccharide (GPPP), and its *in vitro* antioxidant activity was concentration-dependent. At the concentration of 0.9 mg/ml, GPPP had better DPPH radical scavenging ability (91.03%), reducing ability (0.63), and iron ion reducing ability (0.34 mmol/l).

### Hypoglycemic and hypolipidemic

Studies have reported that soy polysaccharides can lower blood sugar and blood lipids, and reduce the risk of diabetes (3). In the study of the effects of soybean polysaccharides on postprandial blood glucose in non-insulin-dependent diabetes mellitus, it was found that soybean polysaccharides could significantly reduce postprandial blood glucose and triglyceride levels (50). Similarly, feeding high-fat diet rats with soybean oligosaccharides at different doses showed that soybean oligosaccharides significantly reduced abnormal blood sugar and blood lipid levels in animal models at all doses (51). Soybean oligosaccharides can reduce the oxidative stress induced by high-fat diet, improve the level of dyslipidemia, and reduce the occurrence of cardiovascular and cerebrovascular diseases.

### Gastrointestinal protection

A large number of studies have proved that plant polysaccharides have activities such as protecting the gastrointestinal tract, regulating intestinal flora, and preventing colorectal cancer (52). Xie et al. (10) investigated the effect of mung bean peel polysaccharide (MBP-2) on the intestinal flora and short-chain fatty acids (SCFAs) in mice. The study found that MBP-2 can increase the colon length and the production of SCFAs in mice, and improve the intestinal microenvironment and promote the growth of beneficial bacteria through SCFAs. Besides, Akhtar et al. fermented chickpea chitin polysaccharides (CHPS) by mimicking human gut flora (53). Studies have shown that CHPS can significantly increase the beneficial gut microbiota and elevate SCFAs.

### Other

Except as mentioned above biological activities, other biological activities of legumes polysaccharides were also evaluated. Hu et al. (16) isolated a novel galactomannan (BSCP-1) with a molecular weight of 7.55× 10^5^ Da from the seed coat of black bean, and found that it had an inhibitory effect on human gastric cancer cells. Moreover, Jiang et al. compared the effects of different extraction methods on the antibacterial activity of mung bean skin polysaccharides (21). The study found that the enzyme-extracted polysaccharide (MBP-1) and the hot-water extracted polysaccharide (MBP-2) showed good antibacterial activity against both Gram-positive bacteria (G^+^) and Gram-negative bacteria (G^−^).

## Functional properties

Legumes polysaccharides also have excellent stability and emulsifying properties, and are mainly used in food and medicine products to improve product stability and emulsifying properties of oil droplets, and significantly improve their quality (3).

For example, two components HMF and LMF with different molecular weights were isolated from soybean polysaccharide. Studies have shown that HMF exhibited high ability to emulsify oil droplets and stabilize α-casein dispersions in acidic environments, and LMF has higher anti-emulsifying lipid oxidation activity (11). Moreover, the functional properties of polysaccharides depend on the ability of the polymer to absorb oil and the interaction between water and polysaccharides (12). It was found that the mung bean water-soluble polysaccharide (MBP-1) had good hydration properties and oil holding capacity (24). Similarly, polysaccharides (CHPS) extracted from chickpea shells have good fat-binding, foaming and emulsifying properties (47).

## Conclusions and perspectives

Like proteins and nucleic acids, polysaccharides also play important roles in living organisms. Bean polysaccharide is the main active ingredient in beans, and is widely used in biology, medicine and functional food due to its excellent functional property and biological activity. In this paper, the extraction, separation and purification, structural properties, biological activities and functional properties of soy polysaccharides are reviewed. Although the research on legumes polysaccharides has made significant progress, there are still many problems to be solved in terms of the current research results. First of all, the extraction and purification method of soybean polysaccharide is inefficient, and there is currently a lack of industrialized and large-scale preparation methods. Second, the structure-activity relationship of soy polysaccharides has not been fully elucidated. Third, regarding the potential risks or toxicity of soy polysaccharides and their derivatives, comprehensive *in vitro* and *in vivo* toxicological studies or risk assessments are required.

## Author contributions

YZ, XF, JG, and XZ contributed to conception and design of the study. YZ wrote the first draft of the manuscript. LW and XG wrote sections of the manuscript. LW, XG, and XZ contributed to funding of the study and writing-review and editing. All authors contributed to the article and approved the submitted version.

## Funding

This work was supported by the National Key R&D Program of China (2021YFD1600100), the earmarked fund for China Agriculture Research System (CARS-08-G21), the National Natural Science Foundation of China (82204668), the Science and Technology Research Project of Higher Education in Hebei Province (QN2020233), and the Agricultural Science and Technology Innovation Program of Chinese Academy of Agricultural Sciences.

## Conflict of interest

The authors declare that the research was conducted in the absence of any commercial or financial relationships that could be construed as a potential conflict of interest.

## Publisher's note

All claims expressed in this article are solely those of the authors and do not necessarily represent those of their affiliated organizations, or those of the publisher, the editors and the reviewers. Any product that may be evaluated in this article, or claim that may be made by its manufacturer, is not guaranteed or endorsed by the publisher.
